# A Word about Proprietaries

**Published:** 1906-04

**Authors:** 


### EDITORIAL NOTES AND COMMENTS.

The Business office of THE JOURNAL-RECORD is No. 1007-1008 Century Building.

The Editorial office is 318-319-320 The Prudential.

Address all Business communications to Dr. M. B. Hutchins, Mgr.

Make remittances payable to THE ATLANTA JOURNAL-RECORD OF MEDICINE.

On matters pertaining to the Editorial and Original Communications address Dr. Bernard Wolff, Atlanta.

Reprints of original articles will be furnished at cost price. Requests for the same should always be made *on the manuscript.*

We will present, post-paid, on request, to each contributor of an original article, twenty (20) marked copies of THE JOURNAL-RECORD containing such article.

### A WORD ABOUT PROPRIETARIES.

It is popular with the profession nowadays to express righteous indignation at the hold which proprietaries have obtained in the matter of prescription writing. The strong plea is made to stick to the old materia medica and give only those good old things which have been tested at the bedside of those who have gone to their reward, and those who are still awaiting it as the years roll over and on. This conservatism and adherence to the old forms is an excellent principle that applies to many things, but is it altogether appropriate to what we are proud to term a progressive art, or even a progressive science? A great many of the so-called proprietaries do no violence to this principle, but even improve upon it. They do not seek to supplant the older materia medica, but to harmonize it with modern usage. Many of the combinations put upon the market by ethical drug houses are merely old and familiar drugs prepared with accuracy, harmoniously grouped and made palatable. The last quality represents one of the chief advances in modern materia medica. Take, for example, cod-liver oil. Who would have the temerity nowadays to prescribe the crude oil, stinking as only a raw and ancient fish can, and this to be given in tablespoonful doses to a patient whose pampered appetite can scarcely be stirred by the most alluring products of the culinary art? In this condition cod-liver oil is not a reconstructive and tonic, as we learn in the books of therapeutics, but a direct and efficient emetic. Now contrast this nauseous draught with the palatable, agreeably smelling emulsion, which contains all the essential qualities of the fish oil, but in such a form as entirely rids it of its objectionable characters in the crude state. If good results are to be expected from remedies, the remedies must be given in such a manner that they may reach the objective point without causing all sorts of disturbances on the way. This is not only true of cod-liver oil, but of many other valuable drug substances of disagreeable odor or taste, which have been rendered capable of being broadly and effectively used by having the objectionable qualities removed or disguised by virtue of special preparation. This preparation is not infrequently a proprietary, and condemned as such.

In addition to palatability, accuracy of dosage is another claim set up by medical specialty manufacturing concerns as being one of their recommendations to favor. This is a matter of great importance, from the point of view of therapeutic results. Many druggists make their own tinctures and fluid extracts, and who knows what is the proper dose of the resultant? It depends entirely upon the druggist's craftsmanship, experience, sense of honor and commercial honesty, and these qualities are personal matters and subject to variation. Some of these fluid extracts and tinctures are not unlike boarding-house coffee, alleged, rather than established. Yet we are not permitted by the unwritten code to recommend any special druggist, and when we give a prescription for a combination of drugs to a confiding patient, we have but little warranty in feeling that it will always be filled according to specifications. An instance somewhat in point, and of interest to the profession as well as to the patient, was that in which a teaspoonful of tincture of aconite was given by a relative to a sick child. When the mistake was discovered several hours had elapsed, and as no harm was done the child the alarm of the family was quelled. If no results followed the administration of a drachm of this particular tincture, it is interesting to all but homoepaths to ponder upon the effects of a therapeutic dose of one or two drops. An incident like the above shakes our confidence in some things connected with the practice of medicine, and makes some of us who are possessed with the New England conscience, feel a self-conviction of obtaining money under false pretences. Now if high-grade proprietaries possess the qualities of palatability and reliability in dosage, it looks from where we sit as if they have some claim upon the respectful attention of the medical profession. There are, of course, many of the so-called proprietaries which, in their claims and their "professor-instructing" type of literature, are an insult to the profession, but there are, at the same time, many others which are genuinely helpful, and should be taken advantage of by every modern and progressive doctor. Automobiles are creeping into great favor among physicians, who assert that they greatly facilitate the operation of their avocation, and yet automobiles are the prince of proprietaries. If there is a consistent prejudice on the part of the medical profession against the principle of proprietaries of any kind and all kinds, as applied to their avocation, the dictum of falsus in uno, falsus in omnibus, must be set forth, and the fast machine must yield to the jogging old Dobbin of yore.

We are not writing in the interest of the proprietaries for hope of fee or reward. Do not look askance at this with any such suspicion. But it is rather fatiguing to hear so much in condemnation of what is really and genuinely meritorious, and often with the knowledge that those who are the most vociferous in denunciation are frequently secret offenders.

